# Using modified whole-mount *in situ* hybridization to study *mpo* expression in zebrafish

**DOI:** 10.3892/etm.2013.947

**Published:** 2013-02-05

**Authors:** LI-JING SHEN, LAN-FANG CAO, FANG-YUAN CHEN, YONG ZHANG, JI-HUA ZHONG, HUA ZHONG

**Affiliations:** 1 Departments of Pediatrics, Shanghai Jiao Tong University School of Medicine, Shanghai 200001, P.R. China; 2 Hematology, Renji Hospital, Shanghai Jiao Tong University School of Medicine, Shanghai 200001, P.R. China

**Keywords:** zebrafish, myeloperoxidase, *in situ* hybridization, myelopoiesis, modify

## Abstract

In this study, we cloned the myeloperoxidase (*mpo*) gene of zebrafish and prepared a digoxigenin-labeled *mpo* RNA probe to investigate *mpo* gene expression in zebrafish during embryonic development by whole-mount *in situ* hybridization (WISH). The earliest *mpo* expression was detected in cells of the intermediate cell mass (ICM) at 18 h post-fertilization (hpf). It was detected 1 to 2 h later in cells in the rostral blood island (RBI) and strong signals were observed in the anterior ICM. Then, it spread over the yolk sac. By 72 hpf these *mpo*-expressing cells were in the circulation and distributed throughout the embryo. We identified that the level of *mpo* expression detected by WISH at an early stage was consistent with the data of cytological analyses of adult fish. The use of this method enabled us to track the gene changes that took place before morphological phenotypes were detected, as well to as investigate the hematopoietic cell fate in mutational or transgenic models *in vivo*. In this study, we modified several steps of WISH. The improved hybridization results demonstrated high specificity, distinct coloration and low background figures.

## Introduction

In the last decade, the zebrafish (*Danio rerio*) was identified as a new genetic system in which to analyze hematopoietic development. Hematopoiesis in zebrafish is similar to that in mammals and other higher vertebrates whose representative blood cell types include the erythroid, thrombocytic, myeloid and lymphoid lineages. In mammals, primitive hematopoiesis occurs outside the embryo, in the blood islands of the yolk sac. Later in development, it moves to the aorta-gonadmesonephros (AGM) region, fetal liver and ultimately the bone marrow. By contrast, primitive hematopoietic stem cells (HSCs) in the zebrafish embryo are born intra-embryonically in ventral mesoderm-derived tissue called the intermediate cell mass (ICM). During this wave, the anterior part of the embryo generates myeloid cells, while the posterior part generates mostly erythrocytes and a number of myeloid cells. From 24 h post-fertilization (hpf), these primitive blood cells start to circulate throughout the embryo. Subsequently, the definitive HSCs emerge from the ventral wall of the dorsal aorta and migrate to the posterior region in the tail called the caudal hematopoietic tissue (CHT). From 3 days post-fertilization (dpf), lymphopoiesis initiates in the thymi. By 4 dpf, HSCs seed the kidney marrow, which is equivalent to bone marrow in mammals (1).

Myelopoiesis is the process of producing all types of myeloid cells including granulocytes and monocytes/macrophages. A number of zebrafish myelopoietic genes are expressed in patterns consistent with their mammalian orthologs, including myeloperoxidase (*mpo*), an enzyme that is a major component of human neutrophil and eosinophil granules. It is also a marker for zebrafish granulocytes (2). Myeloid cells have a wide spectrum of activities, including immune surveillance and tissue remodeling. Irregularities in myeloid cell development and their function are associated with the onset and the progression of a variety of human disorders, including leukemia (3). An in-depth study of *mpo* expression and function in zebrafish is likely to improve our ability to identify, isolate and culture hematopoietic cells to enhance our ability to use this simple organism to address disease biology.

Whole-mount *in situ* hybridization (WISH) is the method of choice to characterize the spatial distribution of gene transcripts during embryonic development. Initial protocols used non-radioactive digoxigenin (DIG)-labeled probes that permit for the first time visualization of global gene expression patterns in *Drosophila* embryos (4). We present a modified protocol, using a DIG-labeled probe to detect the spatio-temporal spectrum of *mpo* expression in zebrafish, which reduces the number of steps and obtains signal enhancement.

## Materials and methods

### Animals

The AB zebrafish strain was maintained at 28.5°C as described by Westerfield (5). Embryos were staged as described by Kimmel *et al* (6). Developmental stages refer to hpf or dpf. This study was approved by the Institutional Animal Care and Use Committee (IACUC) of Shanghai Research Center for Model Organisms (Shanghai, China) with approval ID 2012-0008.

### Experimental materials

Various restriction enzymes and the TOP10 *Escherichia coli* strain were purchased from Takara Bio Inc. (Japan). T4-DNA ligase was purchased from Promega Corporation (Madison, WI, USA) and DNA high fidelity polymerase KOD-Plus was purchased from Toyobo Co., Ltd. (Japan). The DIG-deoxyribonucleotide triphosphate (dNTP) labeling kit, blocking reagents, anti-DIG-AP and BM purple were purchased from Roche Diagnostics (Indianapolis, IN, USA). RNase inhibitor was purchased from Ambion (USA), levamisole, heparin and yeast RNA were purchased from Sigma (St. Louis, MO, USA). TRIzol reagent was purchased from Invitrogen Life Technologies (Carlsbad, CA, USA) and the plasmid Maxiprep kit was purchased from Qiagen (Hilden, Germany). The first-strand cDNA Quantscript RT kit was purchased from Tiangen Biotech Co., Ltd. (Beijing, China). A fluorescence microscope (SMZ-1500; Nikon, Japan), ultra-violet spectrophotometer (ASP-3700; ACT Gene, Piscataway, NJ, USA), Biometra T personal polymerase chain reaction (PCR) amplification instrument (Goettingen, Germany), gel imaging analysis system (Tanon 3500, Shanghai, China) and SPX biochemical incubator (GNP-9160; Shanghai Jinghong Laboratory Instrument Co., Ltd., China) were also used.

### Cloning and mpo/pBK-CML plasmid construction

Total RNA was extracted from 40 embryos at 48 hpf, using TRIzol reagent according to the manufacturer’s instructions. The cDNA was synthesized from 1 *μ*g total RNA using the first-strand cDNA Quantscript RT kit. The specific primers were designed according to the zebrafish *mpo* (*zmpo*) genomic sequence on the National Center for Biotechnology Information (NCBI) web site (gene ID, 337514); forward primer: 5’-TTCAAGTCCAGAACCAGTGAGCCT-3’ and reverse primer: 5’-TTTAGCAGTGGCAGGAAGGATGGA-3’. The length of the amplified PCR product was 2442 bp, including two restriction enzyme cutting sites (*Xho* I at 216 bp and *Eco*RI at 2384 bp) and the probe sequence (at 737 bp). The probe sequence was designed to span exon borders of the gene. PCR was performed as follows: 95°C for 10 min; then 35 cycles at 95°C for 30 sec, 58°C for 30 sec and 72°C for 60 sec. The integrity of the PCR product was examined by 1% agarose gel electrophoresis. The purity was analyzed based on the absorbance ratio at 260 and 280 nm (A260/280). Then the *zmpo* fragment and the pBK-CML vector were digested with *Xho*I and *Eco*RI enzymes and connected at the same sticky end with the T4 ligase, which resulted in the construct of an *mpo*/pBK-CML plasmid.

### Plasmid linearization and probe incubation

The *mpo*/pBK-CML plasmid was linearized with the *Sal*I restriction enzyme. DIG-antisense RNA probes were generated by T7 *in vitro* transcription (1 *μ*g linearization template DNA, 1 *μ*l DIG-dNTP mix, 2 *μ*l 100 mM dithiothreitol (DTT), 4 *μ*l 5X transcription buffer, 1 *μ*l RNase inhibitor, 1 *μ*l T7 RNA polymerase and diethylpyrocarbonate (DEPC) H_2_O to make 20 *μ*l, incubated for 2 h at 37°C). Then, 1 *μ*l DNaseI was added and incubated at 37°C for 20 min to purify the product. The final precipitation was stored at −20°C.

### Embryo preparation

The required developmental stage of the embryos was selected according to the somite. Embryos younger than 48 hpf were dechorionated. The embryos were digested in 1 mg/ml pronase for ∼2–10 min. The digestion was stopped when >10% embryos were free from their chorions. The chorions were broken with air from a pipe. If the chorions were difficult to remove, they were manually broken with a pair of tweezers. Embryos older than 24 hpf were decolored in 5% hydrogen peroxide and 5% potassium peroxide fish water for 15 min. Embryos (up to 40 embryos per 1.5 ml eppendorf tube) were fixed in fresh 4% paraformaldehyde (PFA) in phosphate-buffered saline (PBS) and agitated overnight at 4°C. Then, the embryos were washed twice in PBS with Tween-20 (PBST) for 5 min each and dehydrated with gradient (25, 50, 75 and 100%) methanal/PBST for 10 min each at room temperature (RT), then stored at −20°C.

### First day of hybridization

The embryos were removed from the −20°C storage and rehydrated with gradient (75, 50, 25 and 0%) methanal/PBST. The embryos were fixed with 4% PFA for 20 min and rinsed twice with PBST for 5 min at RT. The embryos were digested with an appropriate concentration of proteinase K (10 *μ*g/ml in PBST) at 37°C for 3 min and then fixed and rinsed as above. They were then incubated at 68°C in negative hybrid liquid (HYB^−^, 50% formamide in 5X SSC buffer) for 15 min and prehybridized at 68°C in 300 *μ*l positive hybrid liquid (HYB^+^, 0.5 mg/ml yeast RNA and 50 *μ*g/ml heparin in HYB^−^) for 4 h. Following this, the HYB^+^ was replaced with fresh HYB^+^ containing the DIG-labeled probe (concentration, ∼1 ng/*μ*l) and incubated overnight at 68°C.

### Second day of hybridization

The probe was removed and the embryos were washed twice for 30 min each with 50% formamide in 2X SSC buffer, then for 15 min in 2X SSC buffer and twice for 30 min each in 0.2X SSC buffer at 68°C. Then, the embryos were rinsed three times for 5 min each at RT in MABT (100 mM maleic acid, 150 mM NaCl and 0.1% Tween-20; pH 7.5) and blocked for 1 h at RT with blocking solution (10% blocking reagent and 50% lamb serum in MAB). Anti-DIG-AP (1:4000 dilution in blocking solution) was added and agitated overnight at 4°C.

### Third day of hybridization

The embryos were washed with blocking solution for 30 min and MABT for 1 h at RT. Then, the embryos were soaked three times for 5 min each in staining buffer (100 mM Tris, 50 mM MgCl_2_, 100 mM NaCl, 0.1% Tween-20 and 1 mM levamisole; pH 9.5). The embryos were transferred to a 12-well plate, incubated in 1 ml/well BM purple stain for 30 min, monitored with a dissecting microscope every 30 min. The reaction was terminated by being washed twice in PBST and fixed with 4% PFA at 4°C to store. Images were taken in 3% methyl cellulose.

## Results

### mpo/pBK-CML plasmid construction and probe synthesis

**Figure 1:**
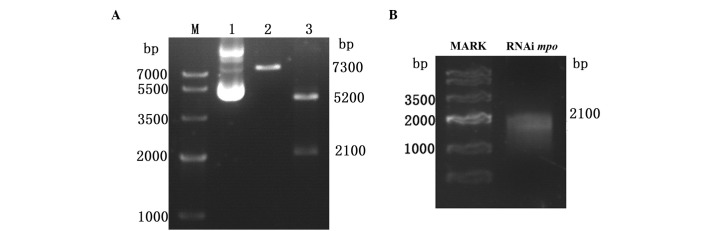
Electrophoresis map of recombinant plasmid and *mpo* probe. (A) Electrophoresis map of the *mpo*/pBK-CML recombinant plasmid. M, Tiangen marker IV; lane 1, plasmid crude extract. Three bands were observed; relaxed DNA, open circle plasmid and superhelix plasmid; lane 2, the plasmid was linearized by *Sal*I enzyme, which obtained the single band at 7300 bp; lane 3, two enzymes, *Xho*I and *Eco*RI, obtained two bands at 5200 and 2100 bp, respectively, which are the pBK-CML vector and the *mpo* gene segment. (B) Electrophoresis map of the antisense *mpo* probe. The band was at 2100 bp. *mpo*, myeloperoxidase.

The cDNA of 48 hpf embryos was used as a template to amplify the *mpo* gemonic fragment by PCR. The PCR product was verified by 1% agarose gel electrophoresis and the single band at 2400 bp was observed as expected. The PCR product was cloned into the pBK-CML carrier and the *mpo*/pBK-CML recombinant plasmid was constructed. The plasmid was linearized by *Sal*I and the single band at 7300 bp was observed. The plasmid was digested with two enzymes (*Xho*I and *Eco*RI), producing two bands at 5200 and 2100 bp, respectively, which were the pBK-CML vector and *mpo* gene segment. This demonstrated that plasmid construction was successful (Fig. 1A). The DIG-labeled antisense *mpo* mRNA probe was generated by T7 *in vitro* transcription and confirmed in the electrophoresis tank soaked with DEPC H_2_O overnight. There was a single band near 2100 bp (Fig. 1B).

### Temporal and spatial expression patterns of mpo

**Figure 2:**
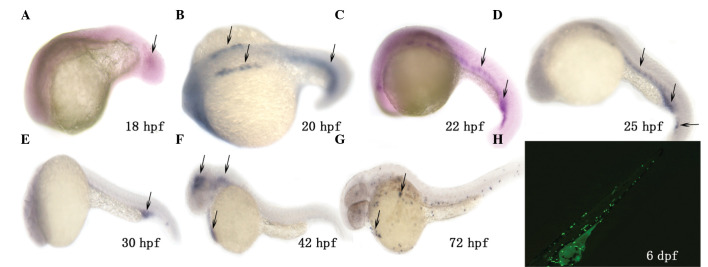
Spatio-temporal spectrum of *mpo* expression in the AB zebrafish strain. *In situ* hybridization with a digoxigenin-labeled RNA probe was used to detect zebrafish *mpo* expression in embryos at 18 hpf (A), 20 hpf (B), 22 hpf (C), 25 hpf (D), 30 hpf (E), 42 hpf (F) and 72 hpf (G). Black arrows indicate *mpo* expression in the intermediate cell mass (ICM) (A-E), rostral blood island (RBI) and pericardial cavity (F). At 72 hpf these *mpo*-expressing cells were in the circulation and distributed throughout the embryo (G). (H) Distribution of fluorescent neutrophils (*mpo*^+^) in our previously established Tg(*zlyz*:EGFP) embryo at 6 dpf. *mpo*, myeloperoxidase; EGFP, enhanced green fluorescent protein; hpf, hours post-fertilization; dpf, days post-fertilization.

The expression patterns of *mpo* were investigated in zebrafish embryos from 12 to 72 hpf by *in situ* hybridization using the DIG-labeled antisense RNA probe. As shown in Fig. 2, the earliest expression of zebrafish *mpo* was detected in cells of the ICM at 18 hpf and 1 to 2 h later, it was detected in cells in the rostral blood island (RBI). Strong signals were observed in the anterior ICM, then it spread over the yolk sac. By 72 hpf the *mpo*-expressing cells were in the circulation and distributed throughout the embryo. In our previous study, we established the transgenic enhanced green fluorescent protein [Tg(*zlyz*:EGFP)] zebrafish line (7), which expresses EGFP in primitive neutrophils (*mpo*^+^). The EGFP distribution coincided with the result of *in situ* hybridization of *mpo* (Fig. 2).

### In situ hybridization and cytological analyses

**Figure 3:**
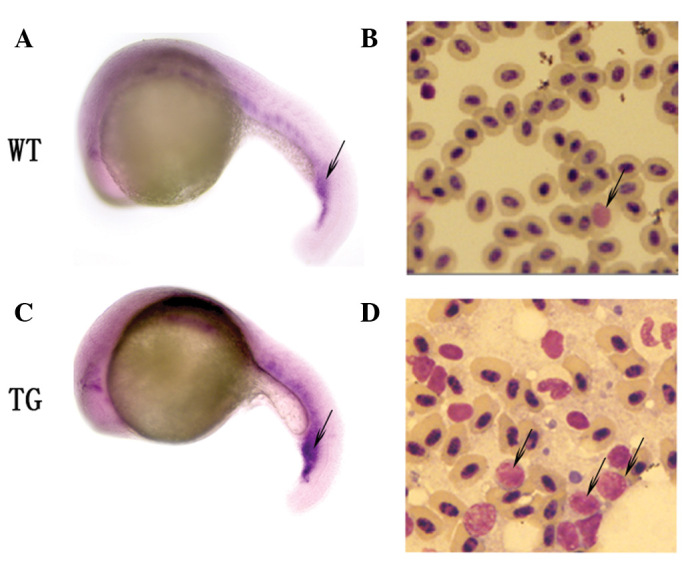
*In situ* hybridization of *mpo* expression and peripheral blood smear comparison between wild type and Tg(*MYCN*:HSE:EGFP) zebrafish. *In situ* hybridization with a digoxigenin-labeled RNA probe to detect wild type (WT) (A) and our previously established Tg(*MYCN*:HSE:EGFP) (TG) (C) zebrafish *mpo* expression in embryos at 22 hpf. Black arrows indicate greater *mpo* expression in TG zebrafish than in WT embryos. Cytological analyses of hematopoietic cells from WT (B) and TG (D) zebrafish at 60 dpf. Blood cells from WT fish were predominantly erythrocytes, with myeloid cells occasionally observed. By contrast, erythrocytes were significantly inhibited in TG fish, which were enriched with granulocytes (indicated by black arrows). *mpo*, myeloperoxidase; EGFP, enhanced green fluorescent protein; TG, transgenic; hpf, hours post-fertilization; HSE, heat shock elements.

To test whether *mpo* expression detected by *in situ* hybridization at an early stage could predict the myelopoiesis in zebrafish development, we compared the peripheral blood smear between wild type (WT) and Tg zebrafish. In our previous study, we established the Tg(*MYCN*:HSE:EGFP) zebrafish line, which suggested that *MYCN* converted erythropoiesis to myelopoiesis (Shen *et al*, the influence of MYCN gene on the transcriptional regulation of hematopoiesis. abs. 751, 9–12 June, 2011). As demonstrated by *in situ* hybridization, the *MYCN* gene increases the expression of *mpo* in embryos at 22 hpf (Fig. 3A and C). For cytological analyses, blood cells collected from the zebrafish at 60 dpf were transferred onto glass slides using Cytospin, stained with Wright-Giemsa stain and examined under oil immersion by light microscopy (8). This revealed that the blood cells from WT fish were predominantly erythrocytes, with myeloid cells occasionally observed. By contrast, erythrocytes were significantly inhibited in Tg fish, which were enriched with granulocytes (Fig. 3B and D).

### Modified WISH

In order to avoid background staining, unincorporated nucleotides were removed from the probe preparation. We routinely used Ambion NucAway Spin Columns to purify the RNA probe according to the manufacturer’s instructions (Cat. AM10070). Following the final precipitation, the hydrolyzed probe was placed in HYB^+^ (final concentration, ∼1 ng/*μ*l) and stored at −20°C. Each time the probe was used, it was degenerated in advance by placing it at 95°C for 5 min then on ice for 5 min to eliminate single RNA spontaneous formation of hairpin structures. Probes were reused for 4–5 times. When 24 hpf embryos were collected, the conventional decolorization method was used and they were placed in 0.03% phenylthiourea (PTU) in fish water at 12–36 hpf. We used the improved decolorizing liquid of 5% hydrogen peroxide and 5% potassium peroxide in fish water. As a result, the embryos only required soaking at 24 hpf for 10–15 min. The effect of decolorization was good without damaging the integrity of the embryo. Prior to hybridization, the embryos were permeabilized by digesting with proteinase K (10 *μ*g/ml in PBST) at RT for 5–30 min, depending on the developmental stage. The permeability of the probe was appropriate and the staining background was low. In addition, the traditional permeabilization method included digesting the embryos at RT for 5–30 min, depending on the developmental stage. This is difficult to control. We improved the digestion temperature to 37°C and the process was shorted to 5 min.

## Discussion

The zebrafish system has a number of unique advantages compared to other vertebrate model organisms. The embryos are externally fertilized and transparent, enabling *in vivo* visualization of early embryonic processes ranging from birth of HSCs in the mesoderm to migration of blood cells. In addition, large production of embryos makes phenotype-based forward genetics feasible (9). A steadily increasing number of hematopoietic-specific genes have been cloned in zebrafish, providing molecular reagents and markers for specific stages of hematopoietic differentiation and specific cell types. We constructed the DIG-labeled *mpo* RNA probe to investigate *mpo* gene expression in zebrafish embryos. The earliest *mpo* expression was detected in cells of the ICM at 18 hpf and 1–2 h later, it was detected in cells in the RBI. Strong signals were observed in the anterior ICM, then it spread over the yolk sac. By 72 hpf the *mpo*-expressing cells were in the circulation and distributed throughout the embryo, with a tendency for a subpopulation of *mpo*-expressing cells to be aggregated in the ventral vein region. Later-stage expression was difficult to distinguish by WISH.

We identified that the level of *mpo* expression detected by WISH at an early stage was consistent with the result of cytological analyses of adult fish (Fig. 3). This method enabled us to track the gene changes that took place before morphological phenotypes were detected, as well as investigate the hematopoietic cell fate in mutational or transgenic models *in vivo*. Given the considerable morphological and functional parallels between zebrafish and mammalian myeloid cells, it is not surprising that zebrafish show conservation of the molecular regulation of myelopoiesis and the molecular tools for myeloid cell function (3).

In this study, we modified several steps of WISH. We carried out several pretreatment steps to ensure the purity and concentration of the probe, as well as shorten the digestion time by proteinase K at higher temperatures. In the decolorization step, we avoided using PTU, which simplified the zebrafish embryo culture steps and enhanced the environmental protection. Finally, we selected BM purple dye to simplify the staining step. The improved hybridization results demonstrated high specificity, distinct coloration and low background figures.

Using WISH in zebrafish, we have the ability to identify and study the lesions of myelopoiesis. Therefore, the powerful genetic approaches applicable in this model, the genomic resources being collected by the international zebrafish and genomic communities and the ability to study myeloid development in this model organism provide new insights into the myeloid arm of developmental hematology.
